# Consumption: Its Nature, Etc.

**Published:** 1886-02

**Authors:** 


### BOOK REVIEWS.

CONSUMPTION: ITS NATURE, CAUSES, PREVENTION AND CURE. *By* J. M. W. KITCHEN, M. D., *Assistant Physician to the Bellevue Chest Class* (*O. D. Dept.*); *Assistant Surgeon to the Metropolitan Throat Hospital; late instructor in Diseases of the Nose and Throat at the New York Post-Graduate Medical School; Author of "Students' Manual of Diseases of the Nose and Throat;" "Catarrh, Sore-Throat, Hoarseness," etc.* G. P. PUTNAM'S SONS, *New York, 1885.*

The book begins with some general remarks on consumption, followed by a sketch of the anatomy and physiology of the respiratory organs and their hygiene. Then comes an account of the different forms of consumption; their nature and symptoms; the physical signs; the possibilities of cure, and the causes of the disease. Then follows a discussion of the principles of treatment, and a chapter on the relations of man's surrounding conditions to phthisis, including climate—the atmosphere,—heat and cold,—moisture, light,—locality and soils,—food,—physical culture,—hereditary transmission,—occupation,—dress,—social habits and surroundings,—individual habits,—ventilation and heating. The succeeding chapters are on the office of medicine in the cure of phthisis and the political duty of the people in regard to the disease, after which the book closes with some general remarks.

This extended, though not very systematic plan, covers ground enough for a large treatise instead of a duodecimo of 223 pages. The book is not even compactly written. It is in the highest degree sketchy, and many things that we would naturally expect here are either left out entirely or are passed over so briefly as to be of little value.

There are some sensible, though self-evident remarks on the hygiene of those having phthisis, or who are predisposed to it.

The chapter on treatment is too general for the student and too commonplace for the physician.

The position of the profession with respect to the tubercle bacillus is inaccurately, not to say, unfairly stated. One familiar with the history of its investigation would certainly have written differently.

The part on anatomy and physiology of the lungs might have been as well written by a newspaper reporter.

The author writes fluently, so much so that his pen often outruns his thought.

The book contains nothing new, and is not better arranged or expressed than has often been done before. The reason for its existence, therefore, and why any one should be expected to read it, are mysteries. L. C.

